# Bare Bones Pattern Formation: A Core Regulatory Network in Varying Geometries Reproduces Major Features of Vertebrate Limb Development and Evolution

**DOI:** 10.1371/journal.pone.0010892

**Published:** 2010-05-28

**Authors:** Jianfeng Zhu, Yong-Tao Zhang, Mark S. Alber, Stuart A. Newman

**Affiliations:** 1 Department of Mathematics, University of Notre Dame, Notre Dame, Indiana, United States of America; 2 Center for the Study of Biocomplexity, University of Notre Dame, Notre Dame, Indiana, United States of America; 3 Department of Cell Biology and Anatomy, New York Medical College, Valhalla, New York, United States of America; Center for Genomic Regulation, Spain

## Abstract

**Background:**

Major unresolved questions regarding vertebrate limb development concern how the numbers of skeletal elements along the proximodistal (P-D) and anteroposterior (A-P) axes are determined and how the shape of a growing limb affects skeletal element formation. There is currently no generally accepted model for these patterning processes, but recent work on cartilage development (chondrogenesis) indicates that precartilage tissue self-organizes into nodular patterns by cell-molecular circuitry with local auto-activating and lateral inhibitory (LALI) properties. This process is played out in the developing limb in the context of a gradient of fibroblast growth factor (FGF) emanating from the apical ectodermal ridge (AER).

**Results:**

We have simulated the behavior of the core chondrogenic mechanism of the developing limb in the presence of an FGF gradient using a novel computational environment that permits simulation of LALI systems in domains of varying shape and size. The model predicts the normal proximodistal pattern of skeletogenesis as well as distal truncations resulting from AER removal. Modifications of the model's parameters corresponding to plausible effects of Hox proteins and formins, and of the reshaping of the model limb, bud yielded simulated phenotypes resembling mutational and experimental variants of the limb. Hypothetical developmental scenarios reproduce skeletal morphologies with features of fossil limbs.

**Conclusions:**

The limb chondrogenic regulatory system operating in the presence of a gradient has an inherent, robust propensity to form limb-like skeletal structures. The bare bones framework can accommodate ancillary gene regulatory networks controlling limb bud shaping and establishment of Hox expression domains. This mechanism accounts for major features of the normal limb pattern and, under variant geometries and different parameter values, those of experimentally manipulated, genetically aberrant and evolutionary early forms, with no requirement for an independent system of positional information.

## Introduction

The limbs of vertebrate animals emerge from the embryonic flank as buds of somatopleure-derived mesenchymal cells covered by an epithelial layer, the ectoderm, flattening into paddle shapes as they grow. The most thoroughly studied aspect of limb development is the formation of the skeleton, an array of jointed bone or cartilage elements having a stereotypical pattern that has sustained only modest alterations over the course of evolution [1], [2]. The mechanism of limb skeletal pattern formation is incompletely understood. There is broad agreement, however, concerning the cellular and molecular-genetic interactions underlying the differentiation of the cartilage tissue that forms the embryonic primordia of the bony skeleton of tetrapod limbs and the endoskeleton of fish fins (see [3], [4] for reviews).

**Figure 1:**
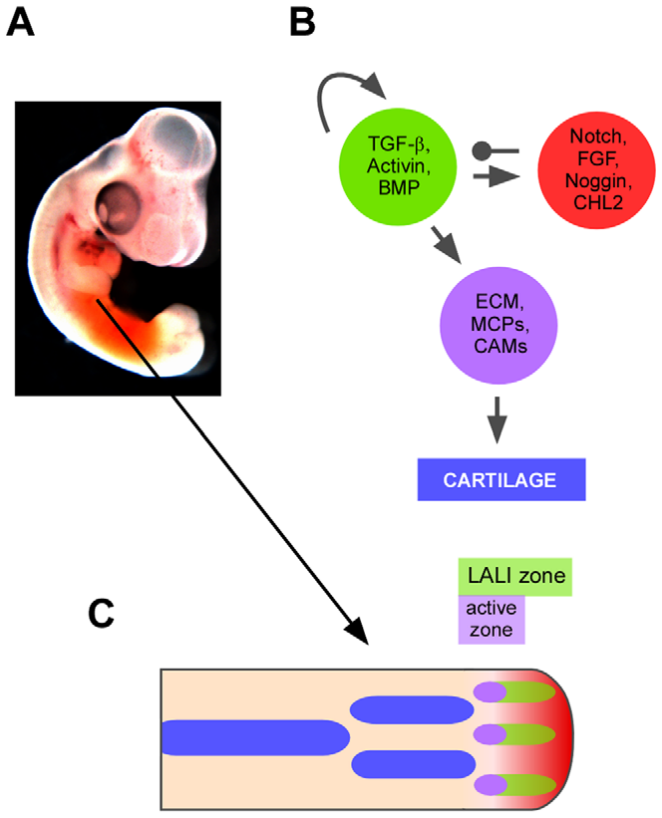
Relationship between core cartilage patterning network and “bare bones” framework for limb development. (A) Chicken embryo at 5 days of development with right fore-limb bud accentuated by staining of the embryonic flank with Eosin. (B) Schematic representation of core mechanism of chondrogenic pattern formation, comprising activator subnetwork (*A*, green circle), inhibitory subnetwork (*I*, red circle) and adhesive, matricellular and ECM molecules induced by *A* (violet). The molecules in the violet circle promote precartilage condensation, which induces chondrogenesis. Subnetwork *A* has positively autoregulatory properties; it also induces *I*, which in turn inhibits *A*. (C) Schematic representation of “bare bones” limb development model in a 2D template corresponding to a 5-day chicken wing bud. The template is divided into a LALI (lateral autoactivation-lateral inhibition) zone in which the reaction-diffusion process defined by *A* and *I* operates. Action of *A* within the LALI zone is suppressed by a diffusible signal (FGF, red-to-pink gradient), with its source at the AER (apical ectodermal ridge) at the distal end (right). At the low end of the FGF gradient a portion of the LALI zone, termed the active zone, is permissive for the production of the cartilage-promoting molecules shown in (B) (violet). Cartilage (blue) forms in cells that leave the LALI zone due to elongation of the limb bud.

A major question regarding limb development concerns how the number and positioning of skeletal elements along the proximodistal (P-D) and anteroposterior (A-P) axes is determined [4]. There is a general P-D increase in the number of skeletal elements which occurs even in cases, such as the chicken forelimb (Fig. 1), in which the A-P width remains essentially constant while the skeletal pattern is being laid out. In fish, non-tetrapod vertebrates, the fin endoskeleton is a mixture of bars and nodules which have no discernable P-D numerical trend [5].

Cartilage differentiation, or chondrogenesis, is preceded by “condensation” of the precartilage mesenchyme, in which cell density increases and the cells enter into broad, transient contact with one another [6]. The precartilage cells are embedded in a dilute extracellular matrix (ECM) and condensation is accompanied by and dependent on local accumulation of the ECM molecule fibronectin [7], with markers of prospective condensation appearing earlier than ECM and morphological changes [8]. Molecules secreted by the dorsal and ventral ectoderm, including FGFs and Wnt, inhibit chondrogenesis [8], [9], thereby confining the developing one-bar proximal cartilage primordium (stylopod, i.e., humerus, femur) to a central planar sector of the paddle-shaped limb bud [4]. As development proceeds, the skeleton remains confined to this plane but expands laterally in more distal regions as the stylopod gives way to the two-bar (zeugopod, i.e., radius and ulna, tibia and fibula) and multiple-bar (autopod, i.e., digits) primordia of the mid and terminal regions of the limb (Fig. 1C). This brings the developing skeletal elements increasingly closer to the anterior and posterior edges of the limb bud, reflecting attenuation of the peripheral inhibitory effect. Attenuation of inhibition can also be seen in the proximity of the more distal elements to the dorsal and ventral surfaces as the limb bud tapers towards its tip, and to the apical boundary, as the potency of the AER wanes [10].

There is a large literature that treats limb pattern formation as a question of establishing spatiotemporal informational fields that cells subsequently interpret according to their genetic programs [11], [12]. Other approaches to this problem (and those of other developing systems), take into account the conditional, self-organizational properties of interacting cells and tissues (see refs. [13] and [14] for reviews). This latter approach has the advantage over the “positional information” perspective in its ability to formulate hypotheses for why the limbs (or other embryonic structures) assume the forms they actually do.

A mechanism that has gained increasing attention as a generator of spot- and stripe-like patterns in developmental systems is related to the chemical reaction-diffusion process described by Turing in 1952 [15], [16]. This has been schematized in a biological “local autoactivation-lateral inhibition” (LALI) framework by Meinhardt and Gierer [17]. LALI systems, while formally similar to reaction-diffusion systems, are more suitable to biological applications, since they include cases where the activator and inhibitor terms are mediated by cellular “reactors” rather than simple chemical reactions [18], and spatial transport can be mediated by mechanisms in addition to simple diffusion [19]. LALI systems are self-organizing: that is, with appropriate choices of activator-inhibitor and transport parameters, in domains of appropriate size, shape and boundary conditions, a spatially uniform distribution of the morphogens becomes dynamically unstable, giving way to nonuniform distributions, typically periodic arrangements of stripes and spots in two dimensions, and their three-dimensional analogs (bars, nodules) [13], [20].

The developing limb bud contains all the components of a skeletogenic LALI mechanism [21], [22], [23]. The locus of activity of this process is a population of multipotent skeletogenic progenitor cells (termed “precartilage” cells in what follows) [24], [25], [26], which are maintained in a non-terminally differentiated state in the distal-most 0.3 mm of the avian and mammalian limb bud by the action of the apical ectodermal ridge (AER), a narrow A-P-oriented ectodermal thickening, along the limb bud tip [27]. Whereas the precartilage cells can give rise to cartilage, connective tissue, and bone, and can undergo programmed cell death, the limb musculature is formed by a separate population of cells [26]. The non-myogenic limb mesenchyme thus forms the skeletal pattern by being caused to choose among its potential fates of cartilage, soft connective tissue fibroblasts and apoptotic cells, in a spatiotemporally regulated fashion.

Precartilage condensation and subsequent chondrogenesis are promoted by morphogens of the TGF-β superfamily [28], [29] that form an “activator subnetwork” (Fig. 1B). In brief, one or more TGF-βs and activins set off a train of events whereby BMPs (one or more of BMP2, 4 and 7), acting via the receptor BMPR-Ia, induce regions of high BMP signaling activity, marked by phosphorylated Smads [30]. Other classes of molecules, such as galactose-binding lectins (“galectins”) [31], may also be involved in this initiation step. Signaling by the activator subnetwork induces extracellular matrix, matricellular and cell adhesion molecules, promoting mesenchymal condensation and subsequent chondrogenesis [32], [33] (Fig. 1B).

The spatial profile of BMP action is dictated not by its receptor distribution, which is uniform, but by a “prepattern” (i.e., a molecular pattern prefiguring the cellular one) of the diffusible morphogen itself, which in the autopod takes the form of narrow crescents at the tips of the forming digits [30]. This prepattern arises from the spatiotemporal dynamics of the activator network morphogens interacting with inhibitory factors. The latter include the extracellular antagonistic BMP binding partners Noggin, Gremlin and Ventroptin (CHL2), the BMP receptor antagonist BAMBI and the antagonistic intracellular co-receptor Smad6, the expression of which are all induced by the activator subnetwork [30], [34]. Consistent with this, the prospective digits and interdigits are uncommitted as to their fates late into autopod formation [30], and this plasticity likely obtains in the fields of precartilage cells that form the stylopod and zeugopod at earlier stages. In particular, exogenous TGF-β can cause noncondensing limb bud precartilage mesenchyme to condense and form ectopic cartilage in vitro [28] and in vivo [34].

The FGF pathway [8] and the Notch pathway [35], [36] are involved in restricting the expansion of precartilage condensations as they form. Along with the inhibitory factors described above, these perinodular chondrogenesis-restricting components constitute what we refer to as the “inhibitor subnetwork” (*I*) (Fig. 1B).

The activator (*A*) and inhibitor (*I*) subnetworks together constitute a core network for the regulation of chondrogenesis (Fig. 1B). All cells of the precartilage mesenchyme appear to be equally capable of producing all components of *A* and *I*. Subnetwork *A* functions in a paracrine and (in part via its TGF-β component [29]), a positively autoregulatory fashion. It induces the production of cell-to-cell transmissible components of subnetwork *I*, which in turn limit subnetwork *A*'s radius of action (Fig. 1B).

Limb bud mesenchyme can in fact self-organize into cartilage patterns with limb-like features. Dissociated and reaggregated limb mesoderm cells, packed into an ectodermal hull, form limb-like arrays of rod-like and nodular skeletal elements [37]. In high-density culture these cells form spots and stripes of cartilage with dimensions and spacing similar to the in vivo patterns [38]. When the physical properties of the culture microenvironment [39] or putative LALI components are manipulated in vitro and vivo, the pattern changes are predictable from LALI models [8], [28], [29], [30].

**Figure 2:**
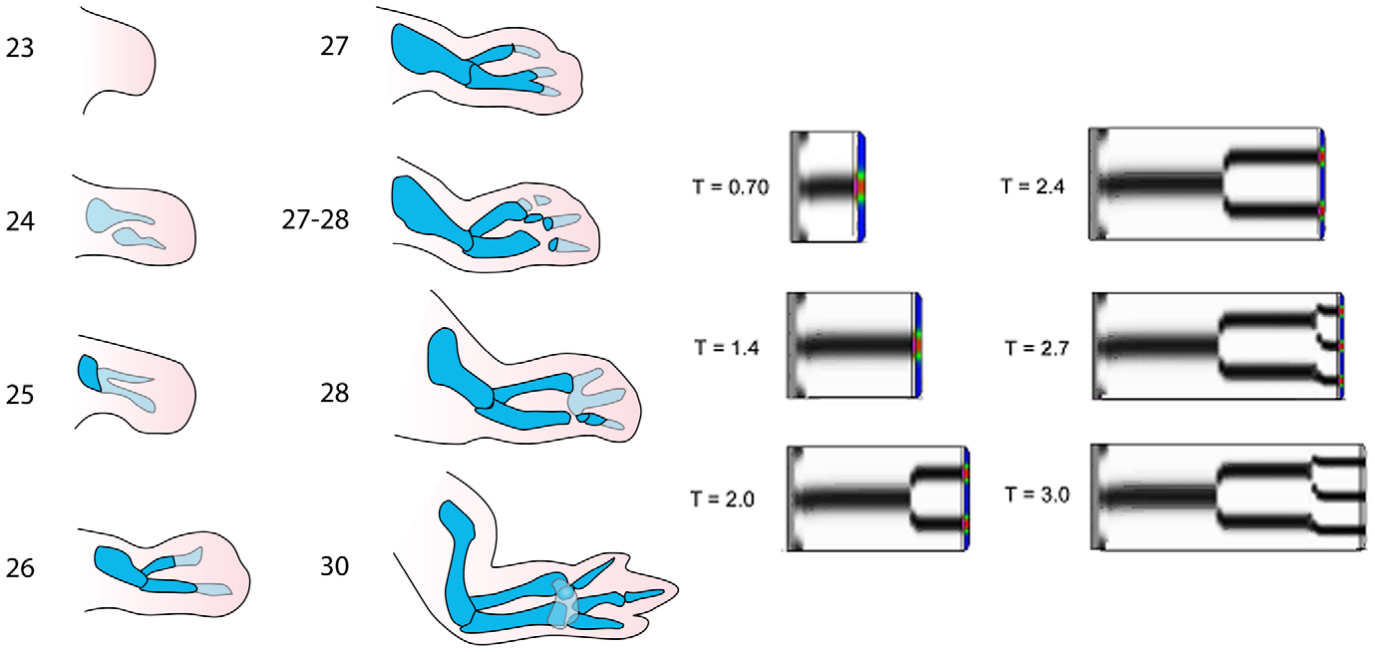
Simulation of chicken wing development. (Left) Developmental progression of chicken forelimb between days 3 and 7 of development (indicated by the corresponding Hamburger-Hamilton stages). Early cartilage, including precartilage condensations, shown in light blue; definitive cartilage shown in darker blue. Based on ref. [23]. (Right) A sequence of snapshots from the simulation of normal limb development. See File S1 for the “standard” set of parameters used. Time is in arbitrary units, but can be used for comparison between different simulations.

In most amniote species, emergence of the cartilage primordia of the limb skeleton occurs first in proximal regions of the limb bud and then in increasingly more distal regions [40] (Figs. 1; 2, left). This process is dependent on an intact AER, the removal of which leads to truncations of the skeleton with progressively more distal elements forming the longer the AER remains in place [40]). The growth of the skeletal structures (and portions thereof) that are spared after AER removal is unaffected, with these components attaining nearly their normal size and shapes, except for terminal deletions, as development proceeds [40]. The limbs of certain amphibians seem to represent partial exceptions to the proximal-to-distal rule [41].

Although the idea that a LALI mechanism could account for major features of limb development in its normal, genetically and experimentally perturbed, and evolutionary aspects is several decades old [23], what has been lacking for most of the succeeding period has been a fast computational modeling environment that would enable simulation of a LALI system as a continuous (“real-time”) process, in domains with natural contours and changing geometries. There has been progress in this direction more recently, using finite element algorithms, producing modeling environments suitable for investigating time- and shape-dependent changes in patterns generated by reaction-diffusion systems of biological interest [42], [43]. Here we use one such framework to test the capability of a LALI limb model to generate realistic patterns under normal and biologically altered conditions.

Below, we analyze the dynamics of a mathematical representation of a limb LALI system in the context of changing domains of realistic proportions, under conditions that take into account uncertainties in the values of parameters such as biosynthetic and diffusion rates and interaction strengths. We show that the contextualized LALI mechanism accounts for major features of the normal limb pattern as well as those of experimentally manipulated, genetically aberrant and evolutionary transitional forms with no a priori requirement for an independent system of positional information.

## Results

### Mathematical representations of the limb skeletogenic system

Simulations of simple LALI systems under geometric and growth constraints partially approximating those of developing limbs show that pattern formation of normal and mutant (e.g., polydactyly, human Apert syndrome, mouse *Doublefoot*) limbs is consistent with such mechanisms [22], [44]. These simulations, however, have variously employed highly schematic and ad hoc molecular and cellular representations, and stationary approximations of inherently temporal dynamics [21], [22]. Up till now, unavailability of adequate computational methods has prevented the continuous time-dependent simulation of a realistic LALI-type system for limb development.

The model of Hentschel and coworkers [18] is the most biologically detailed of the LALI mechanisms for limb development that have been considered. It consists of eight coupled partial differential equations that model all of the following: reaction-diffusion dynamics of a network based on a TGF-β-related activator and a FGF/FGF receptor 2-induced inhibitor, diffusion of FGF from fixed and moving sources, TGF-β-induced production of fibronectin, and the short-range movement of cells and their differentiation from uncondensed mesenchyme to condensed mesenchyme, and then into cartilage, including regulatory dynamics of the associated FGF receptor types. While it would be desirable to follow the time evolution of this system in an initially unpatterned 3D domain that mimics the growing limb bud, the complexity of this prospective simulation exceeds the capacity of available methods.

We have simplified this problem using both biologically motivated assumptions and mathematical arguments [45]. According to a recent classification of developmental mechanisms [46], the limb model of Hentschel et al. [18] is “morphodynamic,” since differentiation of new cell types occurs simultaneously with cell rearrangement. Many developmental mechanisms, in contrast, conform to a simpler “morphostatic” scenario, in which cell identity becomes established independently of cell rearrangement. We have assumed for the purposes of this study that the short-range movement involved in precartilage mesenchymal condensation occurs in populations of cells that have already been determined to do so by developmental signals such as the activating and inhibitory morphogens described above. While the morphostatic assumption is difficult to prove experimentally, it is biologically reasonable. Moreover, the mechanistic separation of cell movement and differentiation can be dispensed with in more elaborate modeling strategies as computational power and methods improve.

Employing the morphostatic limit has allowed us to extract a LALI activator-inhibitor subnetwork from the 8-equation system described in ref. [18]:
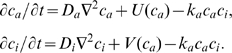
(1)


Here 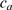 represents the concentration of activating morphogen and 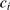 the concentration of inhibitor. The parameters 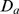 and 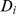 are the respective coefficients of (effective) diffusion, 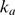 a decay or breakdown constant, and 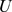 and 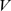 functions that define the production of the morphogens based on the cross- and auto-feedback relationships. Two reaction kinetic parameters incorporated in the functional forms of 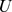 and 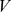, 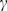 and 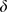 (see ref. [45] and File S1) represent factors that are plausibly altered in value by modulatory transcription factors such as Hox and other gene regulatory proteins [47], and the effects of their variation are described below.

This biologically motivated mathematical simplification of the limb skeletogenic pattern forming system makes it amenable to set of new computational methods for simulation of its time-dependent behavior in regions of arbitrary shape ([42]; also see File S1). In the following sections we describe pattern development in a growing limb-bud-like domain with no imposed structure or pattern other than a simple P-D gradient representing the effect of the AER [27] (Fig. 1C). Our analysis demonstrates that major features of normal, experimentally manipulated, genetically aberrant and evolutionary transitional limb development emerge from the inherent self-organizing properties of the core skeletal patterning mechanism. The roles of factors important in establishing asymmetries in the limb axes and differences among the skeletal elements, such as the morphogen Sonic hedgehog and the Hox gene families (reviewed in ref. [3]), have natural interpretations in this computational framework as regulators of limb bud shape and interaction parameters of the activator-inhibitor network.

### Simulation of normal development

To simulate the developing limb we allowed the LALI system (1) to operate in a geometric setting that represents the section of the growing limb bud equidistant from the dorsal and ventral surfaces (Fig. 1C). A proximodistal gradient of FGF, representing the AER, is initially put in place on this template, with its source at the limb tip. The morphogens are constrained not to leak from the borders of the domain (“no-flux” boundary conditions) and the model limb is programmed to grow uniformly in the P-D direction.

As noted above, the AER keeps precartilage mesenchyme in a labile, undifferentiated state, an effect that has been suggested to be related to its ability to promote outgrowth and distal progression [27], [48]. Moreover, it can be replaced in both these functions by one or more FGFs [49], which diffuse through the distal limb bud mesenchyme [50], [51] forming a proximodistal gradient [52] and regulate cell number and survival [53]. Because the P-D length of the uncondensed distal tip of the chicken limb bud decreases during the period of pattern formation [54] as the potency of the AER wanes [10] we have incorporated into our model the hypothesis that a temporally declining gradient of FGF with its source in the AER determines the P-D length of this distal region, which we term the “LALI zone.” Although the curved distal boundary allows us to simulate the reaction-diffusion dynamics of the LALI zone in more realistic shapes, we did not use the boundary to define a curved source of FGF. Instead the distribution of FGF is modeled as a 1D gradient varying purely along the PD axis, whose value is highest at the distal-most point of the simulation domain. This gradient is represented not by an explicit diffusion equation, but parametrically, by a function that decreases nonlinearly from a maximal value at the distal tip and in which the tip value itself decreases nonlinearly with time (see File S2).

Depending on the geometry of the LALI zone, the activator concentration profile (Fig. 2, right, color-scale) takes the form of one or more elongated spots, analogous to the crescents observed for BMP in vivo [30] (see also Fig. 1C). We define the morphogenetically active region of the LALI zone (the “active zone”; violet bands) as that portion which is at a sufficient distance from the AER to escape the suppressive effect of FGF (see also Fig. 1C). In the full pattern-forming model, cells in the active zone exposed to peak values of activator produce fibronectin and other condensation-promoting molecules [18]. Here we impose a simpler rule by which such cells directly and stably differentiate into cartilage (blue) once elongation of the limb brings them outside the LALI zone. The skeletal elements in this “frozen zone” (with respect to pattern formation) elongate by both growth and addition from newly emerging active zone tissue.

We caused the AER to wane in potency by decreasing the maximal value of the FGF gradient over time. This reduces the P-D length of the LALI zone. Based on the properties of the LALI system (1), this contraction by itself can lead to abrupt increases in the numbers of parallel elements along the A-P axis [45] (see also [18], [23]). We obtained simulation results with a more authentic time-course and proportions (Fig. 2, right; Movie S1) by also changing the parameters 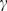 and 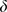 (components of the functions 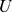 and 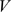) in a stage-dependent fashion (i.e., when the stylopod, zeugopod and autopod are forming) (see File S3). These parameter changes leave the network topology of the reaction-diffusion system unaltered but modify the biosynthetic responses of the morphogens to themselves and each other, and indirectly [45], of ECM production to the morphogens, similarly to demonstrated and proposed functions of the Hox family of transcription factors [47], [55].

Establishment of appropriate expression domains of Hox proteins, some of which change in abundance in the distal mesenchyme in a stage-dependent fashion [56], [57], although not essential for the development of arrays of skeletal elements, are necessary for refined, species-characteristic patterns [58]. Correspondingly, in the parameter range of the standard model the stage-specific variations of the parameters 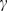 and 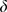 (along with contraction of the LALI zone), are essential to a normal developmental outcome (File S3; see Discussion).

### Robustness of the pattern forming mechanism

**Figure 3:**
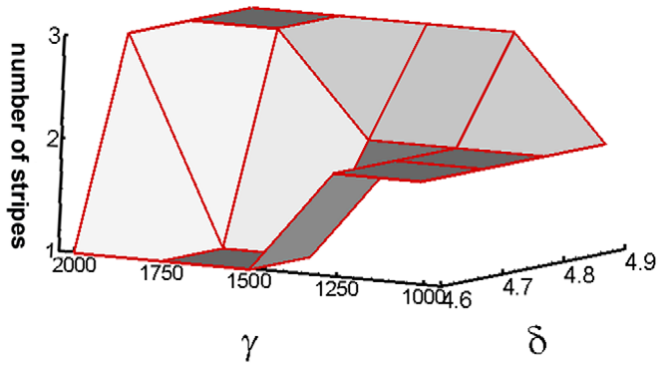
Dependence of the number of stripes on the kinetic parameters 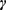 and 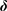 during the first phase of normal development. During this period (0≤T≤1.4, T represents the time), the LALI zone shrinks in the P-D direction (see File S3). One stripe is formed for 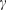 in the range 1500–1750 and 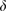 in the range 4.6–4.7, two stripes are formed when 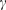 is in the range 1000–1250 and 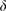 is in the range 4.6–4.8, and three stripes are formed for 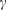 from 1750–2000 and 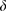 from 4.8–4.9.

Varying key parameters in the LALI system (1) and the geometric properties of the limb simulation template (Fig. 1C) allowed us to explore their effects on the generated pattern and the robustness of the entire process. The reaction kinetic parameters 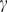 and 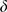 (see ref. [45] and File S1), and the A-P width and P-D length of the LALI zone, are key determinants of the character of the patterns formed. This can be illustrated by the effect of changes in these dimensionless parameters (Fig. 3) in the context of the standard developmental sequence (Fig. 2, right; Movie S1). During the first phase of simulated development (up to 1.4 arbitrary time units), as the LALI zone shrinks in the P-D direction to 2/3 its original length, one (the normal result), two, or three stripes will form when 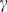 takes on a particular one (±7–11%) of three separated values over a two-fold range, coupled with low, middle or high values (±1%) of 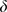 in an overlapping range (Fig. 3).

Based on an extensive set of simulations using altered limb shapes (see below), we have found that the model is much more tolerant to variations in 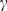 than in 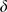. This disparity could be anticipated from the critical role of a narrow range of 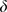 values in the capacity of the system to exhibit pattern formation [45].

The change of parameters in the different phases of the simulation is a plausible computational implementation of the changing distributions of Hox gene products in the apical zone at the different phases of limb development [57]. Hox factors have multiple targets, including genes for cell attachment and ECM molecules, as well as morphogen receptors [47]. Our simulations of the standard developmental progression are sensitive to large and combined changes in parameters 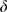 and 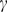, but robust against smaller and individual changes (Fig. 3). For this parameter sweep a single P-D length for the LALI zone was chosen, corresponding to the initial stage of the standard simulation (Fig. 2), when a single stripe is normally specified.

### Simulation of AER removal experiments

**Figure 4:**
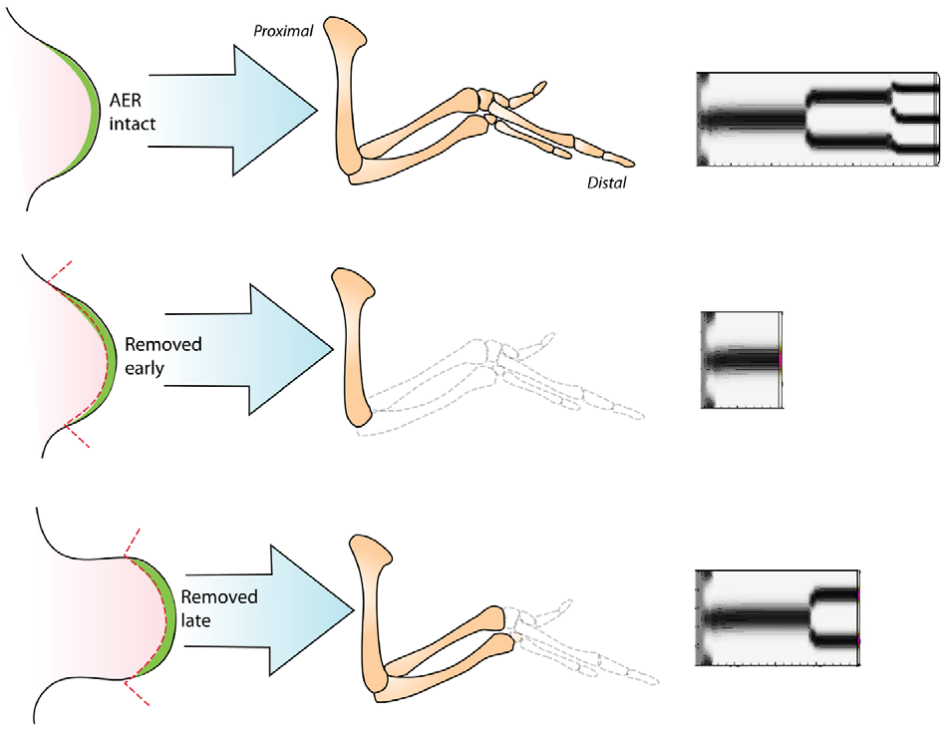
Simulations of AER removal. (Left two columns) Drawings of AER removal experiments, based on Saunders's study [40]. Top images show an intact chicken wing bud at an early stage of development and the limb skeleton that it generates. Middle images show a wing bud at the same early stage with the AER removed, and the resulting limb skeleton, which attains a normal size but is truncated beginning at the elbow. Bottom images show a later stage wing bud whose AER has been removed. The resulting skeleton is truncated from the wrist onward. (Right column) Simulations of limb development using standard parameters. Top: AER (i.e., the source of suppressive FGF morphogen) left intact; normal development results. Middle: AER deleted early during the simulation. Bottom: AER deleted later during the simulation. All simulated limbs were allowed to develop for the same time.

Simulations using the standard parameter set, but with the AER removed part-way during development, show the same result as when this manipulation is performed on developing limbs [40]. In silico, as in vivo, removal of the AER is equivalent to eliminating the distal source of the FGF gradient. After a time lag during which the suppressive gradient decays, the entire LALI zone up to the limb bud tip becomes susceptible to being organized by the activator-inhibitor system, leaving the limb distally truncated (Fig. 4; Movies S2 and S3).

### Simulation of anteroposteriorly expanded limb buds

**Figure 5:**
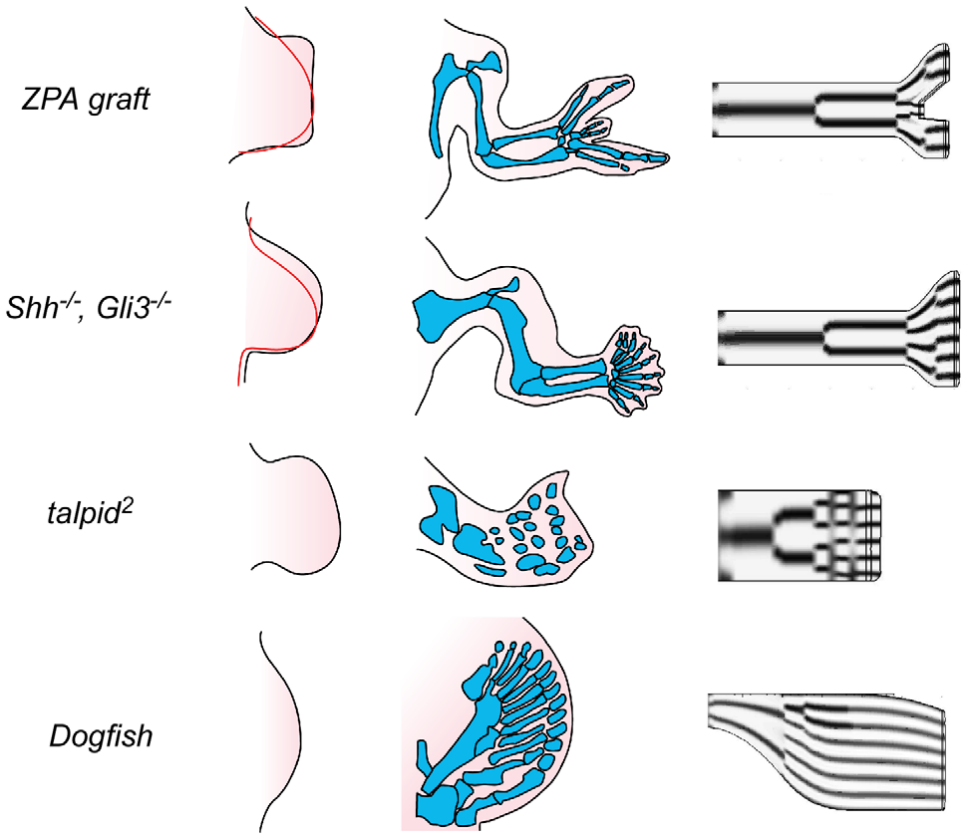
Simulations of effect of limb bud distal expansion. ZPA graft (left) expanded chicken wing bud that results from anterior graft of an ectopic zone of polarizing activity from the proximal posterior region of another wing bud (normal limb profile at this stage shown in red); (center) resulting cartilage skeleton, with mirror-image duplication; (right) end-stage of simulation with distal expansion corresponding to that shown on left. *Shh^−/−^*, *Gli3^−/−^* (left) expanded mouse forelimb bud in embryos null for both *Shh* and *Gli3* (normal limb profile at this stage shown in red); (center) resulting skeleton, with supernumerary digits [61]; (right) end-stage of simulation with distal expansion corresponding to that shown on left. *talpid* ^2^ (left) expanded wing bud of chicken embryo homozygous for *talpid* ^2^ mutation; (center) cartilage skeleton formed from such a limb bud later during development; (right) end-stage of simulation with distal expansion corresponding to that shown on left. Dogfish (left) shape of the pectoral fin-bud in an embryo of the dogfish *Scyliorhinus torazame*; (center) cartilaginous fin skeleton formed from such a limb bud [5]; (right) end-stage of simulation using a limb bud contour like that shown on left. In each of these simulations reaction parameter values different from the standard ones were used (see File S4 for details).

In contrast to the chicken forelimb, many limb buds have an apical zone that expands along the A-P axis during skeletogenic patterning (Fig 5, left). This occurs in the avian hindlimb, in reptilian and mammalian limbs, and to an even greater extent in pathological limbs, such as those of chickens which have received ectopic grafts of the zone of polarizing activity (ZPA) [59], embryos which bear the *talpid^2^* mutation [60], or mice in which the *Sonic hedgehog* (*Shh*) and *Gli3* genes have been jointly knocked down [61]. In the dogfish, while there is no autopod (this being considered an innovation distinguishing fish from tetrapods), a limb bud that is expanded relative to those of birds and mammals produces an array of parallel elements [5], which while not true digits, assume a similar configuration (Fig. 5, left).

The changing limb bud profiles were represented by heuristic equations chosen to emulate plausible reshaping trajectories. Values of 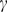 and 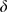 were chosen for 2–7 phases of the continuous simulations, by trial and error to provide a good fit to the final pattern (see File S4).

**Figure 6:**
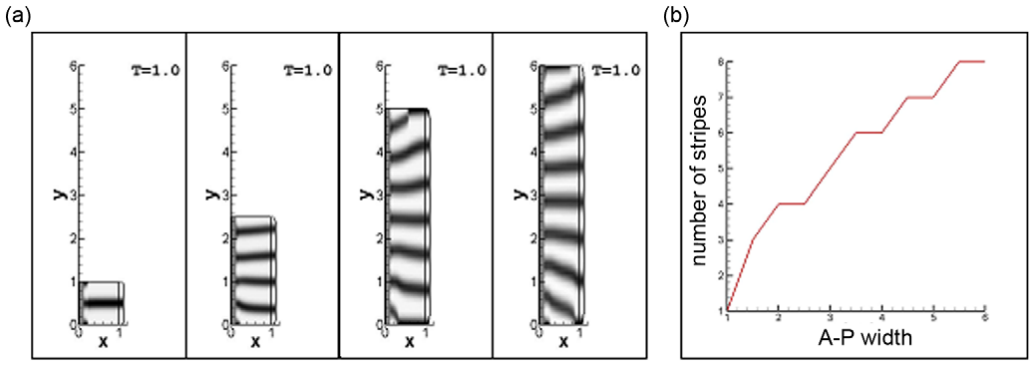
Relation between A-P width and number of stripes. (a) Simulation outcomes. Here 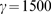 and 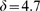. The final time is 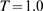. Color legend: black corresponds to 5.5, white to 0.0; (b) graphical representation.

We found that the number of parallel stripes in the A-P direction in the model limb bud increased in a discrete fashion with expansion in the length of the LALI zone, consistent with the general expectation for reaction-diffusion and other LALI systems (Fig. 6). Simulations in which the limb bud or its tip was allowed to expand in a fashion similar to that seen with ZPA grafts into the chicken forelimb, *Shh/Gli3* null mouse embryos, and the chicken mutant *talpid^2^*, show an increased number of parallel “digits” in the autopod (Fig. 5, right; File S4).

### Simulation of fossil limb skeletons

**Figure 7:**
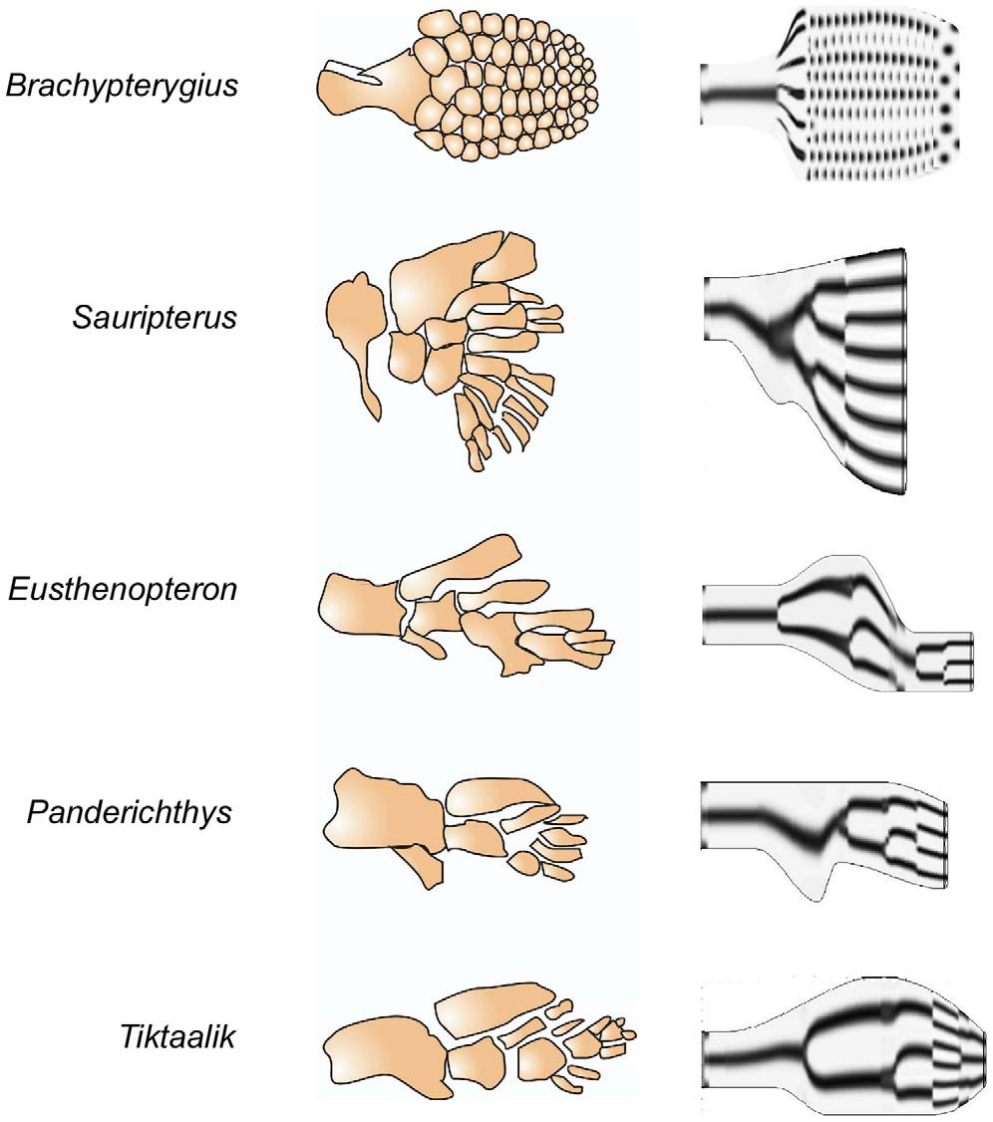
Simulation of fossil limb skeletons. A selection of limb skeletal patterns from fossil specimens (left), were simulated by employing hypothetical developmental scenarios. The end-stages of the simulations of two lobe-finned fish, *Sauripterus* and *Eusthenopteron*, and two forms that are transitional between those organisms and amphibians, *Panderichthys* and *Tiktaalik*, are shown on the right. Snapshots of the full hypothetical sequence for the ichthyosaur *Brachypterygius* are shown in File S5, as are details of the simulations. Drawing of *Brachypterygius* paddle adapted from [63]; drawings of the fossil fish fins based on [62].

Many fossil vertebrate limbs with endoskeletons have been characterized, including some from extinct fish-like ancestors and quasi-tetrapods with authentic autopods [62] (Fig. 7, left). Dinosaur limbs resemble those of modern reptiles and mammals, but ichthyosaurs, swimming dinosaurs, are of interest in that their paddles consist largely of nodular rather than rod-like skeletal elements [63] (Fig. 7, left). We performed simulations based on a wide range of hypothetical developmental scenarios (the one leading to the ichthyosaur-like pattern is shown in File S5) in which the network topology and rules of the standard progression that generated Fig. 2 were maintained, but the contours of the distal region of the limb bud were varied during the simulations (though without prior knowledge of the actual limb bud profiles available for the simulation in Fig. 5), and the parameters 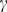 and 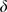 were changed in a stage-specific fashion (see File S5). The simulation end-points shown in Fig. 7 (right) indicate that our model exhibits sufficient flexibility to reproduce the general features of a wide variety of fossil limb skeletons.

## Discussion

The results presented here confirm that a reaction-diffusion process operating in a reshaping domain in the context of a distal suppressive gradient can reproduce major aspects of skeletal patterning in vertebrate limbs. In particular, the proximodistal temporal sequence of development of skeletal elements (Fig. 2), the proximodistally increasing number of elements in fully developed limbs (Fig. 2) and the dependence of development on the suppressive FGF gradient (i.e., the AER), with distal truncation occurring upon its removal (Fig. 4), are all readily accounted for by this mechanism. The “bare bones” patterning mechanism also produces increased numbers of elements when the apical domain is expanded in an anteroposterior direction during development, as occurs with ectopic grafts of the ZPA, with double knockouts of Shh and Gli3, development of the chicken *talpid* ^2^ mutant or the dogfish (Fig. 5).

All of these simulations employed model limbs with curved apical contours, which are truer to the living limb shapes than the simple geometries generally used in reaction-diffusion simulations. The ability to perform such simulations in domains with natural contours and continuously changing nonstandard shapes was enabled by our previous development of new finite element computational methods which are applicable to any biological system in which fields of chemicals or other mobile agents are produced by activator-inhibitor interactions on domains of changing size and shape [42].

By our main hypothesis, limb skeletal patterning is established by a LALI process within a reshaping zone of tissue in the presence of a distal suppressive gradient. While the skeletal patterns in all the limbs we have simulated are mediated by the same self-organizing system, the changing limb bud shapes within which this system operates were imposed arbitrarily according to the schemes described in Files S3, S4, S5. Since limb bud shape is controlled by a different set of molecular determinants from the ones regulating the initiation of chondrogenesis (reviewed in ref. [2]), a more complete model would incorporate an independent system of equations for limb bud shaping [64], [65] involving the growth [66] and viscoelastic [67] properties of the limb bud tissue regulated by molecules such as Wnt, Shh, Gli3, and FGFs [68], [69].

In our model, an increase in the number of parallel elements occurs when either the P-D length of the LALI zone decreases ([45]; first figure of File S3) or its A-P width increases (Fig. 6). While certain other conditions may lead to peak splitting in activator-inhibitor systems [22], [70], we have seen only occasional examples of intercalation of new peaks between preexisting ones with parameter change (e.g., second figure of File S3). Since such patterning modes characterize certain variant limb types [22], this may indicate a limitation of our representation that would improve with the introduction of additional modulatory parameters corresponding to the molecular complexity of the biological system [3], [4], [11].

Joints can form in our model as a result of discontinuities in the pattern of elements along the AP axis as different spatial solutions become stable as the LALI zone changes in size and shape (e.g., Fig. 2; Movie S1). Oscillations are well known to occur in reaction-diffusion system in certain parameter regimes [71], including our morphostatic system (H.G.E. Hentschel and S.A. Newman, unpublished data). Although such oscillations would not generally serve to segment stripe-like LALI pattern elements [70], the elongated elements in our system are typically formed by temporal persistence of spot-like patterns rather than as de novo stripes (e.g., *Brachypterygius* simulation in File S5), making oscillatory modes plausible factors in joint formation. In embryonic limbs the generation of joints depends on members of the BMP superfamily beyond those specified in our core mechanism, such as GDF5 [72]. These factors have activating and inhibitory effects on chondrogenesis that would result in more complex spatiotemporal waves than those seen in the basic model.

In all the simulations shown we have varied the values of the reaction kinetic parameters 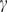 and 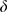 in a P-D level-specific fashion; that is, different values were used for the stylopod, zeugopod and autopod (see Files S3, S4, S5 for details). Although we determined previously that the contraction of the P-D length of the LALI zone (a consequence of the attenuation of the AER suppressive gradient), was by itself sufficient to increase in the number of parallel skeletal elements [45], the variation of 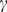 and 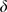 (which does not change the network topology of the core patterning system nor impart any element-specific positional information to the model limb), fine tuned the auto- and cross-regulatory interactions between the morphogens and led, with appropriate choices to relatively authentic skeletal patterns.

The parameters 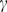 and 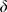 were defined by a detailed mathematical analysis [45] in which factors relating to cell movement and extracellular matrix production were folded into functions (*U* and *V*) governing the activating and inhibitory morphogens operating within a system encompassing a fuller range of biological phenomena [18]. This simplification was necessitated by limitations on the complexity of systems that can be simulated by available finite element algorithms [42]. While attribution of molecular functions to the parameters in the original eight-equation system is more straightforward, it is nonetheless still possible to discern the roles of 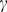 and 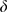 in the morphostatic system (1) and thereby attempt to put a “molecular face” on these parameters.

Both parameters appear in the production rates of the activator and inhibitor morphogens, and 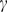 is also the association rate constant of the activator and the inhibitor. The parameter 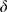 denotes the activator morphogen concentration at which there is a transition from a linear response to a saturation phase. In particular, the Turing bifurcation (a transition in the behavioral characteristics of the system) that enables pattern formation only occurs in a narrow range of 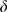 values [45]. This constraint is in evidence in all the simulations shown here (with displacement of 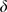
±2% around its mean value significantly affecting the pattern), and can be considered a required relationship for pattern formation among the system's activator and inhibitory components under our biological assumptions and their mathematical representations.

The limb deformity (ld) locus in the mouse encodes several functionalities corresponding to the role of 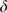 in our model. *Formin1*, disruption of which leads to the absence of the fibula and reduction in digit number, probably regulates expression of BMPs [73], components of the activator subnetwork (Fig. 1b), whereas a transcriptional global control region (GCR) in this domain activates the limb-specific expression of the BMP antagonist Gremlin [74], a component of the inhibitor subnetwork. Not included in the present model, however, are the buffering systems accumulated over 400 million years of evolution that protect the biological equivalent of 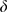 from deviating from its prescribed range of values in present-day limbs.

In contrast to 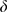, the value of 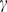 can vary extensively without compromising the pattern-forming capacity of the system. Variations in 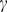 lead to extensive changes in the number and arrangement of skeletal elements, particularly in the autopod, the most variable region of normal, mutant and fossil limbs. As suggested above, the behavior of the system under parameter variation may provide insight into the elusive functions of Hox transcription factors in the developing limb. Different members of the Hoxa family vary in abundance in the limb tip in a stage-dependent fashion [57], and like the parameter 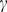, influence activator dynamics. Hoxa13, for example, which is the most highly expressed member in the prospective autopod, alters the expression in the developing limb of two morphogens of the TGF-β superfamily, members of which comprise the activator subnetwork (Fig. 1b) [75]. Hoxa9, abundantly expressed in more proximal regions, modulates TGF-β superfamily signaling [76]. We can thus tentatively identify the role of 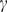 in our scheme with certain functions of the Hoxa gene products.

A different set of Hox gene products, the Hoxd class, vary along the limb bud A-P axis during development [57]. A more detailed model could potentially account for the morphological differences between the radius and ulna, or the various fingers, by parameter variations in this direction as well. As with the dynamical network for limb bud shaping which is not yet included in our framework (see above), equations for setting parameter values in a biological fashion, ultimately based on the Hox gene regulatory network [58], [77], would fill an important gap in the model. It should be noted, however, that such shape and parameter regulatory systems presuppose a core chondrogenic mechanism (Fig. 1B) and a bare bones pattern generating scheme (Fig. 1C), as described above. They do not by themselves provide positional information to the cells of the developing limb.

Despite the extensive sampling of parameter space represented by the simulation results shown, and scores of additional simulations with other parameter combinations, some in which the parameter 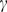 varied by more than 10-fold (Files S3, S4, S5), it was difficult to obtain an outcome that did not resemble a limb skeleton. This in no way means that any arbitrary pattern could be produced: the simulated skeletons are all composed of spot- and stripe-like elements. The LALI system (1), in the context in which we have simulated it, is therefore inherently “skeletogenic.” It appears, moreover, that the topology of the core network, rather than the specific identity of the relevant gene products (of which our knowledge is incomplete, and for which there is variation between tetrapod species), may be decisive for this process.

A recent study has provided evidence that Tiktaalik [78] and other vertebrates known from the fossil record to have noncanonical bony limb skeletons previously thought to be transitional to definitive tetrapod limbs actually coexisted with early tetrapods [79]. The drastically narrowed the time span during which evolution of all known limb morphotypes must have occurred calls for a skeletogenic mechanism with a propensity to generate a profusion of patterns due to small genetic changes affecting limb bud shaping and the rates and strengths of core interactions. The mechanism we propose is the only empirically based one currently under consideration that has these properties.

While our model provides a plausible account of the general form of the limb skeletal pattern (a capability absent in competing models), its main role at present is as a framework for further experimental tests. In particular, it will be important to gain knowledge of the earliest acting activators and inhibitors of precartilage condensation, the details of their transmission and interaction, and the role of FGF or other signals from the AER in defining the morphogenetically active region of the developing limb.

## Methods

Numerical simulations of the LALI system (1) were performed using a new class of finite element algorithms on moving and deforming domains [42], [80] based on the discontinuous Galerkin (DG) method [81], [82]. The DG method is a means for converting an ordinary or partial differential equation system into to a problem represented by a system of algebraic equations in a more restricted space than that of the original system. It employs “independent” polynomials on every element to approximate the system's behavior in the restricted space and provides more flexibility than the continuous Galerkin method. These novel methods enable numerical solutions of system (1) and other LALI and reaction-diffusion systems on deforming and moving grids in domains with complicated geometries. To approximate the irregular geometries of limb buds, we used the cubic spline interpolation technique of de Boor [83]. This method approximates the curved boundary of a limb bud by piece-wise cubic polynomials and maintains global smoothness of the obtained approximation curve.

## Supporting Information

File S1 Description of the partial differential equation model.
(0.07 MB PDF)

File S2 Representation and characteristics of the FGF gradient.
(0.06 MB PDF)

File S3 Dependence of pattern on P-D length of LALI zone and reaction-diffusion parameters.
(0.09 MB PDF)

File S4 Details of limb bud expansion experiments and mutants.
(0.03 MB PDF)

File S5 Details of fossil simulations.
(0.10 MB PDF)

Movie S1 Simulation video of normal development.
(1.77 MB AVI)

Movie S2 Simulation video of early AER removal.
(0.48 MB AVI)

Movie S3 Simulation video of late AER removal.
(1.06 MB AVI)
