# Supplementary material for: Bare Bones Pattern Formation: A Core Regulatory Network in Varying Geometries Reproduces Major Features of Vertebrate Limb Development and Evolution
Source: PLoS One. 2010 May 28;5(5):e10892. doi: 10.1371/journal.pone.0010892 (PMC2878345; doi:10.1371/journal.pone.0010892)
Supplement: File S1 — Description of the partial differential equation model. (0.07 MB PDF) [file pone.0010892.s001.pdf]

Zhu et al., Bare bones pattern formation: a core regulatory network in varying geometries reproduces major features of vertebrate limb development and evolution

File S1: Description of the partial differential equation model

A set of eight coupled partial differential equations was written to represent the following features of the developing limb [1,2]: randomly moving uncommitted precartilage mesenchymal cells produce diffusible activator and inhibitor morphogens (lumped single-variable representations of the activator (*A*) and inhibitor (*I*) networks described in the main text). The activator promotes its own synthesis as well as that of the inhibitor and the inhibitor suppresses the effects of the activator. The cells react to elevated levels of activator by undergoing a reversible transformation to an inhibitor-producing state, from that state to one which produces an adhesive matrix (fibronectin), and then to an immobile state (cartilage). Finally, the system includes a morphogen that, at high levels, keeps cells in the uncommitted state ("FGF"). All morphogens, cell types, and the ECM molecule fibronectin are represented by continuous density variables, and all of them, with the exception of fibronectin and cartilage cells, are considered to diffuse, with characteristic coefficients. As indicated in the main text, this system is referred to as the core patterning network for the developing limb [1,2].

Although the eight-equation system would be infeasible to solve by available analytical or computational methods, it is mathematically "well-behaved" in that smooth solutions for it exist [3]. By considering the restrictive but biologically plausible case in which cell differentiation equilibrates on a faster time scale than the overall density of the mobile cells (the "morphostatic" [4] version of the core mechanism), a two-equation system describing the dynamics of the activator and inhibitor was derived [5]. This system

$$\begin{aligned} \partial c_a / \partial t &= D_a \nabla^2 c_a + U(c_a) - k_a c_a c_i, \\ \partial c_i / \partial t &= D_i \nabla^2 c_i + V(c_a) - k_a c_a c_i, \end{aligned} \qquad (1)$$

thus carries over the original biological framework, including the varying cell densities, along with the additional constraints, in a mathematically explicit and rigorous way. By the morphostatic assumption, cells will differentiate according to the morphogen patterns, and only then arrange themselves into condensed vs. noncondensed regions.

In system (1) c_a denotes the concentration of the activator TGF-β, c_i the concentration of the inhibitor, D_a and D_i the diffusion constants for the activator and the inhibitor respectively, k_a the inhibitor-activator binding rate, and U and V the production rates of c_a and c_i, respectively. The system is subject to no-flux boundary conditions and zero initial concentrations for c_a and c_i. The functions U and V are given by

$$U(c_a) = \left[J_a^1 \alpha(c_a) + J_a(c_a)\beta(c_a) \right] R_{eq},$$
$$V(c_a) = J_i(c_a)\beta(c_a)R_{eq}, \qquad (2)$$

Where

$$J_a(c_a) = J_{a\max}(c_a / s)^n / \left[1 + (c_a / s)^n \right],$$
$$J_i(c_a) = J_{i\max}(c_a / \delta)^q / \left[1 + (c_a / \delta)^q \right],$$
$$\beta(c_a) = \beta_1 c_a / (\beta_2 + c_a).$$

Following [5], the parameter values in the system are taken as $D_a = 1,\ D_i = 100.3,\ J_{a\max} = 6.0\gamma,\ J_{i\max} = 8.0\gamma,\ s = 4.0,\ k_a = \gamma,\ J_a^1 \alpha(c_a) = 0.05\gamma,$ $\beta_1 = 0.693473,\ \beta_2 = 2.66294,\ R_{eq} = 2.0,\ n = q = 2,$

where the values of the reaction kinetic parameters γ, δ which dramatically affect the pattern as shown in [5] and this paper, are varied in a level-specific fashion (see Files S3-S5).

The parameter γ is related to the feedback strength of the activator morphogen, while δ is related to the activator concentration which separates the linear response phase from the saturation response phase in this system. See ref. [5] for additional details on the meaning of these parameters.

References

1. Hentschel HG, Glimm T, Glazier JA, Newman SA (2004) Dynamical mechanisms for skeletal pattern formation in the vertebrate limb. Proc R Soc Lond B Biol Sci 271: 1713-1722.
2. Newman SA, Christley S, Glimm T, Hentschel HG, Kazmierczak B, et al. (2008) Multiscale models for vertebrate limb development. Curr Top Dev Biol 81: 311-340.
3. Alber M, Hentschel HGE, Kazmierczak B, Newman SA (2005) Existence of solutions to a new model of biological pattern formation. Journal of Mathematical Analysis and Applications 308: 175-194.
4. Salazar-Ciudad I, Jernvall J, Newman SA (2003) Mechanisms of pattern formation in development and evolution. Development 130: 2027-2037.
5. Alber M, Glimm T, Hentschel HG, Kazmierczak B, Zhang YT, et al. (2008) The morphostatic limit for a model of skeletal pattern formation in the vertebrate limb. Bull Math Biol 70: 460-483.
