# Supplementary material for: Bare Bones Pattern Formation: A Core Regulatory Network in Varying Geometries Reproduces Major Features of Vertebrate Limb Development and Evolution
Source: PLoS One. 2010 May 28;5(5):e10892. doi: 10.1371/journal.pone.0010892 (PMC2878345; doi:10.1371/journal.pone.0010892)
Supplement: File S2 — Representation and characteristics of the FGF gradient. (0.06 MB PDF) [file pone.0010892.s002.pdf]

Zhu et al., Bare bones pattern formation: a core regulatory network in varying geometries reproduces major features of vertebrate limb development and evolution

File S2: Representation and characteristics of the FGF gradient

We treat the gradient of FGF parametrically, specifying the temporally declining concentration of this morphogen at the distal tip of the limb bud (level A in Fig. S2.1), by a time-dependent nonlinear function. The distal boundary of the active zone, where suppression of cell differentiation by FGF is first relieved, is represented in the model as the boundary (level B in Fig. S2.1) at which the FGF level falls below a fixed concentration (0.4 in arbitrary units). Both the LALI and active zones have their proximal termini at the boundary (level C in Fig. S2.1) at which the FGF concentration vanishes.

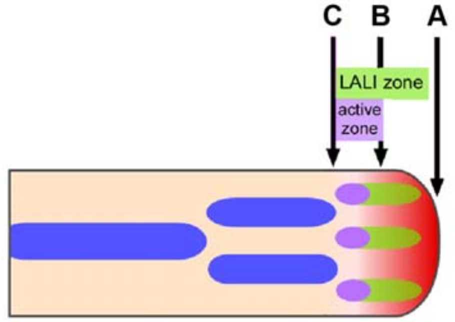

Fig S2.1 Anteroposterior boundary at level A is the distal end of the LALI zone, defined by the position of the AER and the high point of the FGF gradient; boundary at level B is the distal end of the active zone (see text); boundary at level C marks the proximal end of both the LALI and active zones.

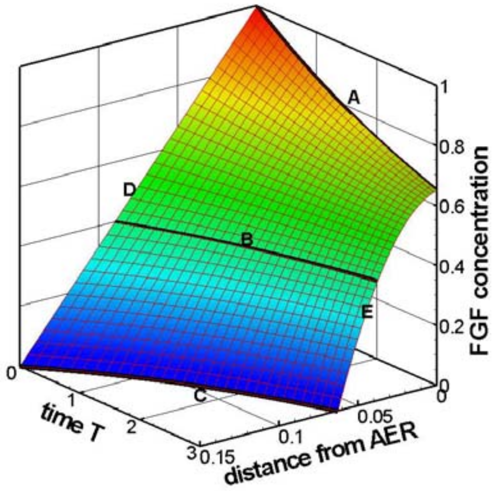

Fig S2.2 Contour drawing of FGF concentration, the value of which depends on the time and the distance from AER. Curve A: FGF concentration at the distal tip of the limb bud (level A in Fig. S2.1); Curve B: FGF concentration at the distal end of the active zone (level B in Fig. S2.1); Curve C: FGF concentration at the proximal end of both the LALI and active zones (level C in Fig. S2.1).

FGF is at its highest concentration value at the AER (level A in Fig. S2.1), but this value decreases nonlinearly with time, described by curve A in Fig S2.2. It satisfies the following formula

$$C_{tip}(T) = 0.4 + 0.6e^{\sigma_x T},$$

where σ_x is a parameter that determines the moving velocity of the LALI zone (see File S3).

The FGF concentration decreases with distance from the distal tip of the limb bud. When the value falls below a fixed value 0.4 arbitrary units, a portion of the LALI zone becomes the active zone at the boundary represented by curve B in Fig. S2.2. Thus, the FGF concentration at the distal end of the active zone (level B in Fig. S2.1) is always 0.4.

The FGF concentration declines further as it approaches the common proximal ends of the LALI and active zones (level C in Fig. S2.1), where the FGF concentration vanishes (curve C in Fig. S2.2). The FGF concentration at this level (C in Fig. S2.1) is always 0.

The FGF concentration profiles across the entire LALI zone at time T=0 and T=3.0 are described by curves D and E in Fig. S2.2. These concentration profiles satisfy the following formula

$$C_{FGF}(T,x) = \frac{4}{0.09}\left(e^{\sigma_x T} - 1\right)e^{-2\sigma_x T}x^2 - \frac{4}{3}\left(8e^{\sigma_x T} - 3\right)e^{-\sigma_x T}x + 0.4 + 0.6e^{\sigma_x T}$$

where T is time and x is the distance from AER. When T is fixed, C_{FGF} depends nonlinearly on x. Thus, neither curve D nor E in Fig. S2.2 are straight lines. Both the distance from level A to level B and from level A to level C in Fig. S2.1 decrease with time (curve B and curve C in Fig. S2.2), which means that the LALI zone is shrinking during limb development. The distance between level B and level C in Fig. S2.1 is 2/3 the distance between level A and level B in Fig. S2.1.
