# Supplementary material for: Bare Bones Pattern Formation: A Core Regulatory Network in Varying Geometries Reproduces Major Features of Vertebrate Limb Development and Evolution
Source: PLoS One. 2010 May 28;5(5):e10892. doi: 10.1371/journal.pone.0010892 (PMC2878345; doi:10.1371/journal.pone.0010892)
Supplement: File S3 — Dependence of pattern on P-D length of LALI zone and reaction-diffusion parameters. (0.09 MB PDF) [file pone.0010892.s003.pdf]

Zhu et al., Bare bones pattern formation: a core regulatory network in varying geometries reproduces major features of vertebrate limb development and evolution

File S3: Dependence of pattern on PD length of LALI zone and reaction-diffusion parameters

In general, a reaction-diffusion or other LALI pattern-forming system will give rise to patterns with characteristics (e.g., peak width and wavelength) independent of domain size and shape. This breaks down for systems in which the ratio of the size of the domain to the critical wavelength of the Turing instability is low [1]. In the system considered here, the shape of the LALI zone alone, without alteration in the reaction-diffusion parameters δ and λ is sufficient to induce substantial changes in the pattern, including changes in wavelength [2]. Change in LALI zone length, accompanied by stage-dependent changes in just one of the parameters, λ, leads to a limb-like [3] progression of 1-2-3 elements in association with narrowing of the apical unpatterned zone of the limb bud (Fig. S3.1).

(a) (b) (c)

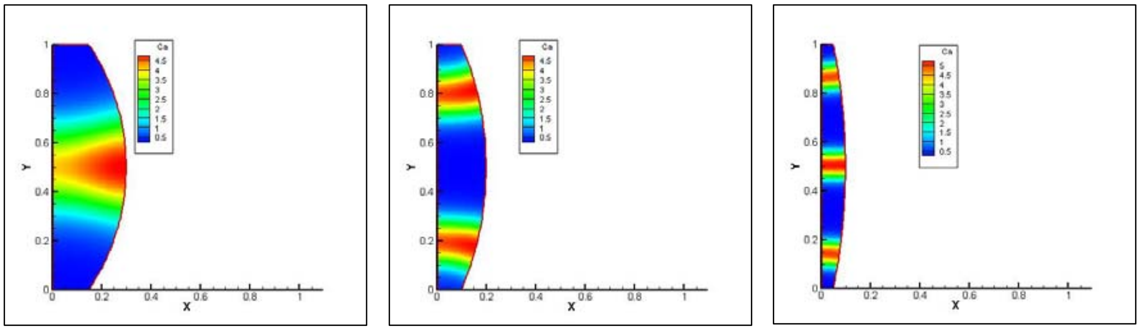

Fig. S3.1 Simulations on a shrinking domain with curved apical zone. (a) $\gamma = 500$, $\delta = 4.7$; (b) $\gamma = 1900$, $\delta = 4.7$; (c) $\gamma = 6900$, $\delta = 4.7$.

Simulation of the Standard Developmental Sequence

There are three color scales in the time-dependent simulation pictures (Fig. 2**)**: grayscale, representing formed cartilage elements of the “frozen zone”; white-violet scale, representing the active zone; and blue-green-red scale, representing the activator morphogen concentration in the apical zone. The LALI zone, in which the reaction-diffusion system is solved, consists of the active zone and apical zone.

At the very beginning the LALI zone comprises the entire limb bud. Since the FGF gradient gets more and more shallow, the LALI zone shrinks in the simulation in the x direction, and the LALI zone growth rate is modeled as $x'(T) = \sigma_x (x - vT) + v$ with $\sigma_x = -0.2896,\ v = 1.0$. It remains the same in the y direction in the simulation, i.e., $y'(T) = 0$. The frozen zone grows at the speed $v = 1.0$. At every 0.05 unit of time, we

copy the concentration values on the computation grids of the left boundary of LALI zone to the new grid points in the frozen zone. The FGF concentration at the very tip of AER is modeled by $C_{tip}(T) = 0.4 + 0.6e^{\sigma_x T}$. The simulation is carried up to time $T = 3.0$. The P-D length of the LALI zone (see Fig. 1C, and Fig. S2.1 of File S2) is represented by $L_{LALI}(T) = 0.15e^{\sigma_x T}$ and the length of the active zone (Fig. 1C and Fig. S2.1 of File S2) is $L_{Active}(T) = 0.4L_{LALI}(T)$.

From time $T = 0$ to 1.4, $\gamma = 1500$ and $\delta = 4.7$. From $T = 1.4$ to 2.4, $\gamma = 5000$ and $\delta = 4.9$. From $T = 2.4$ to 3.0, $\gamma = 16500$ and $\delta = 4.9$. The time step size is $\Delta t = 2 \times 10^{-5}$ in the simulations.

The formation of the normal pattern sequence is dependent both on the stage-dependent changes in λ and δ, which we associate with changes in Hox protein distribution, and on the observed narrowing of the unpatterned apical mesoderm during development (Fig. S3.2).

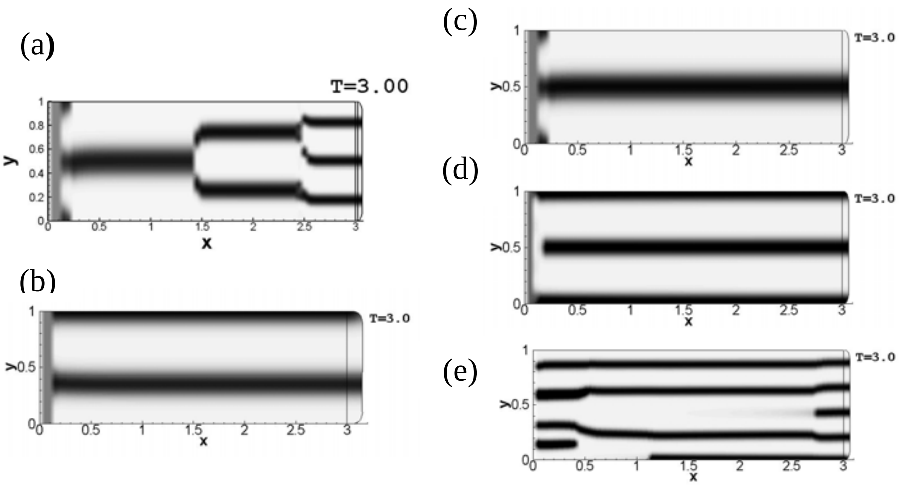

Fig. S3.2 Simulation outcomes in which various constraints of the standard case are relieved. (a) Normal endpoint (from Fig. 2); (b) LALI zone fixed, other parameter changes as normal; (c-e) LALI zone narrows as normal during simulation, but γ and δ values remain unchanged: $\gamma = 1500$ and $\delta = 4.7$ for c, $\gamma = 5000$ and $\delta = 4.9$ for d, and $\gamma = 16500$ and $\delta = 4.9$ for e. Color legend: (a) black corresponds to 6.1, white to 0.0; (b-e) black corresponds to 5.6, white to 0.0.

References

1. Borckmans P, Dewel G, De Wit A, Walgraf D (1995) Turing bifurcations and pattern selection. In: Kapral R, Showalter K, editors. Chemical waves and patterns. Dordrecht; Boston: Kluwer Academic Publishers. pp. 323-363.

2. Alber M, Glimm T, Hentschel HG, Kazmierczak B, Zhang YT, et al. (2008) The morphostatic limit for a model of skeletal pattern formation in the vertebrate limb. Bull Math Biol 70: 460-483.
3. Summerbell D (1976) A descriptive study of the rate of elongation and differentiation of skeleton of the developing chick wing. J Embryol Exp Morphol 35: 241-260.
