# Supplementary material for: Bare Bones Pattern Formation: A Core Regulatory Network in Varying Geometries Reproduces Major Features of Vertebrate Limb Development and Evolution
Source: PLoS One. 2010 May 28;5(5):e10892. doi: 10.1371/journal.pone.0010892 (PMC2878345; doi:10.1371/journal.pone.0010892)
Supplement: File S4 — Details of limb bud expansion experiments and mutants. (0.03 MB PDF) [file pone.0010892.s004.pdf]

Zhu et al., Bare bones pattern formation: a core regulatory network in varying geometries reproduces major features of vertebrate limb development and evolution

File S4: Details of limb bud expansion experiments and mutants

The panels of Fig. 5 display simulations of limbs resulting from ZPA grafting, Shh-Gli3 knockout, *talpid*^{2} embryos and dogfish development. For all but the dogfish, from $T=0$ to 2.4 we have the same setup and same parameter values as the standard development case. The LALI zone shrinks in the simulation in the x direction the same speed as the standard development case, i.e., for node $[x(T),y(T)]$ in LALI zone $x'(T)=\sigma_x(x-vT)+v$ with $\sigma_x=-0.2896, v=1.0$ and $y'(T)=0$ in these three simulations. The upper boundary curve is $\left[x_{upper}(T),y_{upper}(T)\right]=\left[T,1\right]$, and the lower is $\left[x_{lower}(T),y_{lower}(T)\right]=\left[T,0\right]$.

For the Shh-Gli3 knockout simulation (2nd of Fig.4), the shrinking of the LALI zone in the x direction stays the same from $T=2.4$ to 3.0 as the standard development case. We consider the trajectory of the two ending points of the left boundary line of the LALI zone. The upper one moves along the curve

$\left[x_{upper}(T),y_{upper}(T)\right]=\left[T,-6.9444(T-2.4)^3+6.25(T-2.4)^2+1\right]$ from $T=2.4$ to 3.0

and the lower one moves along the curve

$\left[x_{lower}(T),y_{lower}(T)\right]=\left[T,12.5(T-2.4)^3-7.5(T-2.4)^2\right]$ from $T=2.4$ to 2.8 and

$\left[x_{lower}(T),y_{lower}(T)\right]=\left[T,-0.4\right]$ from $T=2.8$ to 3.0.

Then we have explicit speeds y'_{upper} and y'_{lower} for these two ending points at each time from $T=2.4$ to 3.0. Next, considering any point $[x_{left}(T),y_{left}(T)]$ on the left boundary line of the LALI zone, we have

$$y_{left}(T)=\left[y_{left}(0)-y_{lower}(0)\right]\frac{y_{upper}(T)-y_{lower}(T)}{y_{upper}(0)-y_{lower}(0)}+y_{lower}(T)$$

and

$$y'_{left}(T)=\left[y_{left}(0)-y_{lower}(0)\right]\frac{y'_{upper}(T)-y'_{lower}(T)}{y_{upper}(0)-y_{lower}(0)}+y'_{lower}(T).$$

For each node $[x(T),y(T)]$ in the LALI zone, we set $y'(T)=y'_{left}(T)$ for any $y(T)=y_{left}(T)$. Thus, the vertical growth rate of the LALI zone depends on the location of the nodes.

For the ZPA grafting simulation we have same vertical growth rate of the LALI zone as for the Shh-Gli3 knockout. The only difference is that $y'(T)=0$ from $T=2.4$ to 3.0 for the LALI zone nodes whose initial y-locations are in the range $1.0/3\le y(0)\le 2.0/3$, and the frozen zone grows at the speed of $v_1=0.5$, other than $v=1.0$ from $T=2.4$ to 3.0 for

the nodes $[x(T), y(T)]$, where $1.0/3 \le y(0) \le 2.0/3$. Thus, for nodes $[x(T), y(T)]$ where $1.0/3 \le y(0) \le 2.0/3$ in the LALI zone, we have $x(T) = x(0)e^{\sigma_x T} + 2.4v + (T - 2.4)v_1$, and $x'(T) = \sigma_x[x(T) - v_1 T - 2.4v + 2.4v_1] + v_1$. While for other nodes, we have $x(T) = x(0)e^{\sigma_x T} + Tv$, and $x'(T) = \sigma_x[x(T) - vT] + v$ as before. For both the ZPA grafting and Shh-Glii3 knockout, from $T = 2.4$ to 3.0, $\gamma = 20000$ and $\delta = 4.9$.

For the *talpid*^{2} simulation we have the same growth speeds in the x and y directions as the standard development case. The only differences are the values of the key parameters γ and δ. From $T = 0$ to 0.5, $\gamma = 1500$ and $\delta = 4.7$. From $T = 0.5$ to 0.8, $\gamma = 5000$ and $\delta = 4.9$. From $T = 0.8$ to 0.9, $\gamma = 20000$ and $\delta = 4.9$. From $T = 0.9$ to 0.95, $\gamma = 1000$ and $\delta = 4.9$. From $T = 0.95$ to 1.05, $\gamma = 25000$ and $\delta = 4.9$. From $T = 1.05$ to 1.1, $\gamma = 500$ and $\delta = 4.9$. From $T = 1.1$ to 1.2, $\gamma = 25000$ and $\delta = 4.9$. The simulation ends at $T = 1.2$.

In the dogfish fin simulation the trajectory of the upper ending point of the left boundary line of the LALI zone is
$\left[x_{upper}(T), y_{upper}(T)\right] = \left[T, 1.5T^2 + 1\right]$ from $T = 0$ to 1.0,
$\left[x_{upper}(T), y_{upper}(T)\right] = \left[T, T^3 - 6T^2 + 12T - 4.5\right]$ from $T = 1.0$ to 2.0, and
$\left[x_{upper}(T), y_{upper}(T)\right] = \left[T, 3.5\right]$ from $T = 2.0$ to 3.0.

The lower one is
$\left[x_{lower}(T), y_{lower}(T)\right] = \left[T, 0\right]$ from $T = 0$ to 1.5, and
$\left[x_{lower}(T), y_{lower}(T)\right] = \left[T, 0.1481T^3 - 0.6667T^2 + T - 0.5\right]$ from $T = 1.5$ to 3.0.

For the key parameter values, $\gamma = 3000$, $\delta = 4.7$ from $T = 0$ to 1.0, and $\gamma = 13000$, $\delta = 4.7$ from $T = 1.0$ to 3.0. In this simulation, $x'(T) = \sigma_x(x - vT) + v$ and $\sigma_x = -0.4055,\ v = 1.0$. $y(T)$ and $y'(T)$ have the same expression as in the Shh-Gli3 knockout simulation.

In all four simulations shown in Fig. 5, the length ratio between active zone and apical zone is the same as that in the standard development case.
