# Supplementary material for: Bare Bones Pattern Formation: A Core Regulatory Network in Varying Geometries Reproduces Major Features of Vertebrate Limb Development and Evolution
Source: PLoS One. 2010 May 28;5(5):e10892. doi: 10.1371/journal.pone.0010892 (PMC2878345; doi:10.1371/journal.pone.0010892)
Supplement: File S5 — Details of fossil simulations. (0.10 MB PDF) [file pone.0010892.s005.pdf]

Zhu et al., Bare bones pattern formation: a core regulatory network in varying geometries reproduces major features of vertebrate limb development and evolution

File S5: Details of fossil simulations

The full hypothetical developmental sequence for the ichthyosaur *Brachypterygius* is shown below, with the corresponding parameters. For the other fossil forms, the structures of which are shown in Fig. 7, only the parameters are given.

Brachypterygius

The final time is 2.55. Let $x'(T)$ be the speed of the LALI zone and v be the speed of the frozen zone. We have the parameter values:

From T = 0 to 1.5

$$\begin{cases} x'(T) = \sigma_x (x - vT) + v, \\ y'(T) = \left[y(0) - y_{lower}(0) \right] \dfrac{y'_{upper}(T) - y'_{lower}(T)}{y_{upper}(0) - y_{lower}(0)} + y'_{lower}(T), \\ \sigma_x = 0.4621, \quad v = 1.0. \end{cases}$$

From T = 1.5 to 1.75

$$\begin{cases} x'(T) = \sigma_x (x - vT + 1.5) + v, \\ y'(T) = \left[y(0) - y_{lower}(0) \right] \dfrac{y'_{upper}(T) - y'_{lower}(T)}{y_{upper}(0) - y_{lower}(0)} + y'_{lower}(T), \\ \sigma_x = 0.4621, \quad v = 2.0. \end{cases}$$

From T = 1.75 to 2.55

$$\begin{cases} x'(T) = \sigma_x (x - vT + 3.25) + v, \\ y'(T) = \left[y(0) - y_{lower}(0) \right] \dfrac{y'_{upper}(T) - y'_{lower}(T)}{y_{upper}(0) - y_{lower}(0)} + y'_{lower}(T), \\ \sigma_x = 0.4621, \quad v = 3.0. \end{cases}$$

$$\begin{cases} \gamma = 1500, \quad \delta = 4.7, \text{ from } T = 0 \text{ to } 1.5, \\ \gamma = 20000, \quad \delta = 4.7, \text{ from } T = 1.5 \text{ to } 1.75, \\ \gamma = 5000, \quad \delta = 4.7, \text{ from } T = 1.75 \text{ to } 2.55. \end{cases}$$

Since $\sigma_x = 0.4621$ is positive, the LALI zone is not shrinking anymore.

The trajectory for upper ending point is

$$
\begin{cases}
\left[x_{upper}(T), y_{upper}(T)\right] = \left[T, 1\right], \text{from } T = 0 \text{ to } 0.5; \\
\left[x_{upper}(T), y_{upper}(T)\right] = \left[T, 0.1T^3 - 0.05T^2 - 0.025T + 1.0125\right], \text{ from } T = 0.5 \text{ to } 1.5; \\
\left[x_{upper}(T), y_{upper}(T)\right] = \left[2T - 1.5, -10.8(2T-1.5)^3 + 56.2(2T-1.5)^2 - 95.2(2T-1.5) + 54\right], \\
\qquad \text{from } T = 1.5 \text{ to } 1.75; \\
\left[x_{upper}(T), y_{upper}(T)\right] = \left[3T - 3.25, -0.0556(3T-3.25)^3 + 0.4167(3T-3.25)^2 - (3T-3.25) + 2.7778\right], \\
\qquad \text{from } T = 1.75 \text{ to } 2.55.
\end{cases}
$$

And the trajectory for lower ending point is

$$
\begin{cases}
\left[x_{lower}(T), y_{lower}(T)\right] = \left[T, 0\right](), \text{from } T = 0 \text{ to } 0.5; \\
\left[x_{lower}(T), y_{lower}(T)\right] = \left[T, -0.1T^3 + 0.05T^2 + 0.025T - 0.0125\right](), \text{ from } T = 0.5 \text{ to } 1.5; \\
\left[x_{lower}(T), y_{lower}(T)\right] = \left[2T - 1.5, 10.8(2T-1.5)^3 - 56.2(2T-1.5)^2 + 95.2(2T-1.5) - 53\right], \\
\qquad \text{from } T = 1.5 \text{ to } 1.75; \\
\left[x_{lower}(T), y_{lower}(T)\right] = \left[3T - 3.25, 0.0556(3T-3.25)^3 - 0.4167(3T-3.25)^2 + (3T-3.25) - 1.7778\right], \\
\qquad \text{from } T = 1.75 \text{ to } 2.55.
\end{cases}
$$

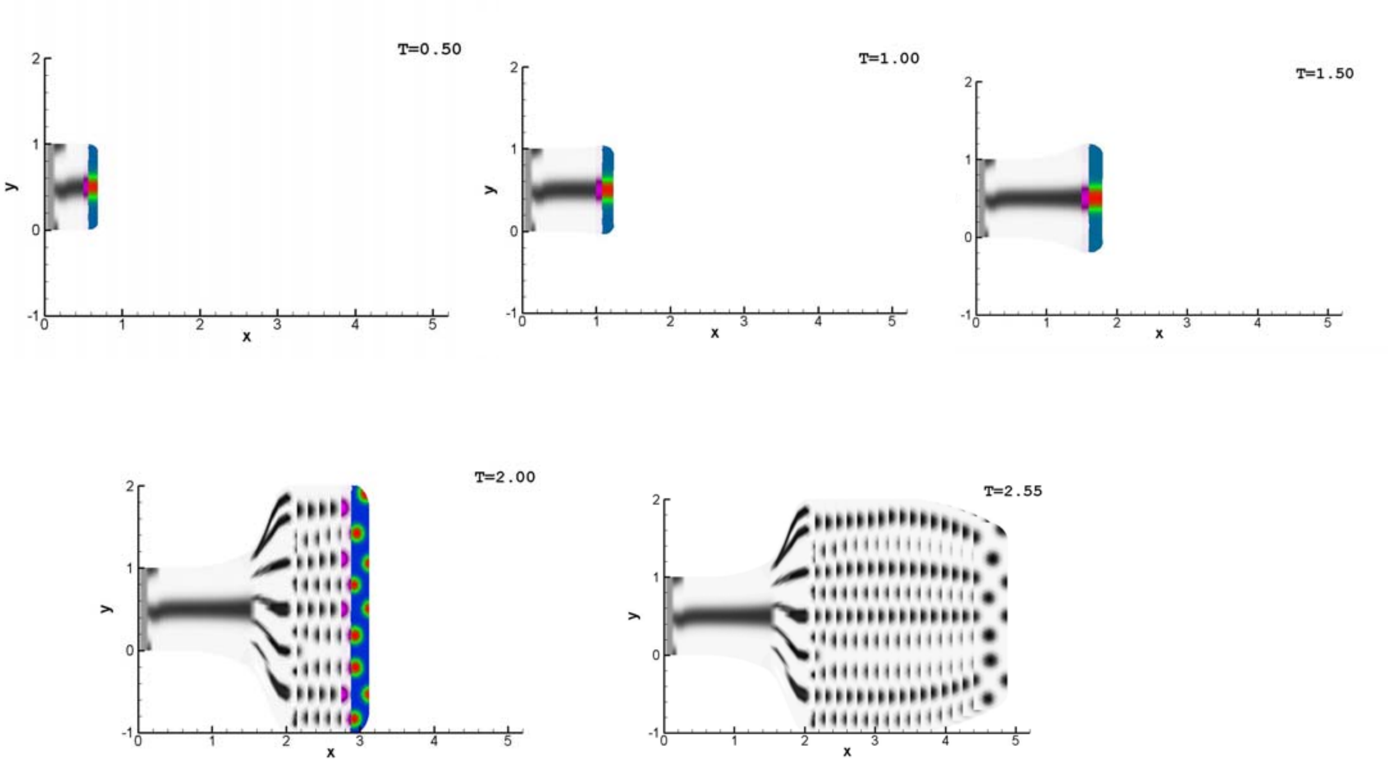

Fig. S5.1 Simulation of hypothetical developmental sequence leading to the limb skeleton of the fossil form of the ichthyosaur *Brachypterygius*. Color legend: black/red/ violet corresponds to 7.0, white/blue to 0.0, green corresponds to the median level 3.5.

Sauripterus

The final time is T=3.50

$$\begin{cases} x'(T) = \sigma_x (x - vT) + v, \\ y'(T) = \left[y(0) - y_{lower}(0) \right] \dfrac{y'_{upper}(T) - y'_{lower}(T)}{y_{upper}(0) - y_{lower}(0)} + y'_{lower}(T), \\ \sigma_x = -0.2027, \quad v = 1.0. \end{cases}$$

$$\begin{cases} \gamma = 1500, \quad \delta = 4.7, \text{ from } T = 0 \text{ to } 0.5, \\ \gamma = 1000, \quad \delta = 4.7, \text{ from } T = 0.5 \text{ to } 1.2, \\ \gamma = 1000, \quad \delta = 4.9, \text{ from } T = 1.2 \text{ to } 1.8, \\ \gamma = 3000, \quad \delta = 4.9, \text{ from } T = 1.8 \text{ to } 2.5, \\ \gamma = 10000, \quad \delta = 4.9, \text{ from } T = 2.5 \text{ to } 3.5. \end{cases}$$

The trajectory for upper ending point is

$$\begin{cases} \left[x_{upper}(T), y_{upper}(T) \right] = \left[T, 1 \right], \text{ from } T = 0 \text{ to } 0.5, \\ \left[x_{upper}(T), y_{upper}(T) \right] = \left[T, -2.5T^3 + 7.25T^2 - 5.375T + 2.1875 \right], \text{ from } T = 0.5 \text{ to } 1.5, \\ \left[x_{upper}(T), y_{upper}(T) \right] = \left[T, -1.5T^3 + 9.75T^2 - 19.625T + 14.5625 \right], \text{ from } T = 1.5 \text{ to } 2.5, \\ \left[x_{upper}(T), y_{upper}(T) \right] = \left[T, 0.1481T^3 - 1.7778T^2 + 7.1111T - 5.9815 \right], \text{ from } T = 2.5 \text{ to } 3.5. \end{cases}$$

The trajectory for lower ending point is

$$\begin{cases} \left[x_{lower}(T), y_{lower}(T) \right] = \left[T, 0 \right], \text{ from } T = 0 \text{ to } 0.5, \\ \left[x_{lower}(T), y_{lower}(T) \right] = \left[T, 0.0233T^3 - 0.1574T^2 + 0.1399T - 0.0335 \right], \text{ from } T = 0.5 \text{ to } 3.5. \end{cases}$$

The simulation result shown for *Sauripterus* in Fig. 7 is inverted vertically relative to the above.

Eusthenopteron

The final time is T=5.00

$$\begin{cases} x'(T) = \sigma_x (x - vT) + v, \\ y'(T) = \left[y(0) - y_{lower}(0) \right] \dfrac{y'_{upper}(T) - y'_{lower}(T)}{y_{upper}(0) - y_{lower}(0)} + y'_{lower}(T), \\ \sigma_x = -0.2027, \quad v = 1.0. \end{cases}$$

$$\begin{cases} \gamma = 1500, \quad \delta = 4.7, \text{ from } T = 0 \text{ to } 1.5, \\ \gamma = 5000, \quad \delta = 4.9, \text{ from } T = 1.5 \text{ to } 2.0, \\ \gamma = 4000, \quad \delta = 4.9, \text{ from } T = 2.0 \text{ to } 3.5, \\ \gamma = 1000, \quad \delta = 4.7, \text{ from } T = 3.5 \text{ to } 4.0, \\ \gamma = 5000, \quad \delta = 4.9, \text{ from } T = 4.0 \text{ to } 4.5, \\ \gamma = 17000, \quad \delta = 4.9, \text{ from } T = 4.5 \text{ to } 5.0. \end{cases}$$

The trajectory for upper ending point is

$$\begin{cases} \left[x_{upper}(T), y_{upper}(T) \right] = \left[T, 1 \right], \text{ from } T = 0 \text{ to } 1.5, \\ \left[x_{upper}(T), y_{upper}(T) \right] = \left[T, -0.2T^3 + 1.5T^2 - 3.15T + 3.025 \right], \text{ from } T = 1.5 \text{ to } 3.5, \\ \left[x_{upper}(T), y_{upper}(T) \right] = \left[T, 1.8 \right], \text{ from } T = 3.5 \text{ to } 5.0. \end{cases}$$

The trajectory for lower ending point is

$$\begin{cases} \left[x_{lower}(T), y_{lower}(T) \right] = \left[T, 0 \right], \text{ from } T = 0 \text{ to } 1.5, \\ \left[x_{lower}(T), y_{lower}(T) \right] = \left[T, T^3 - 6T^2 + 11.25T - 6.75 \right], \text{ from } T = 1.5 \text{ to } 2.5, \\ \left[x_{lower}(T), y_{lower}(T) \right] = \left[T, -0.5 \right], \text{ from } T = 2.5 \text{ to } 3.0, \\ \left[x_{lower}(T), y_{lower}(T) \right] = \left[T, -2.6T^3 + 27.3T^2 - 93.6T + 104.8 \right], \text{ from } T = 3.0 \text{ to } 4.0 \\ \left[x_{lower}(T), y_{lower}(T) \right] = \left[T, 0.8 \right], \text{ from } T = 4.0 \text{ to } 5.0. \end{cases}$$

The simulation result shown for *Eustenopteron* in Fig. 7 is inverted vertically relative to the above.

Panderichthys

The final time is T=4.00

$$\begin{cases} x'(T) = \sigma_x (x - vT) + v, \\ y'(T) = \left[y(0) - y_{lower}(0) \right] \dfrac{y'_{upper}(T) - y'_{lower}(T)}{y_{upper}(0) - y_{lower}(0)} + y'_{lower}(T), \\ \sigma_x = -0.2027, \quad v = 1.0. \end{cases}$$

$$\begin{cases} \gamma = 1500, \quad \delta = 4.7, \text{ from } T = 0 \text{ to } 1.0, \\ \gamma = 1000, \quad \delta = 4.7, \text{ from } T = 1.0 \text{ to } 2.3, \\ \gamma = 3000, \quad \delta = 4.9, \text{ from } T = 2.3 \text{ to } 3.0, \\ \gamma = 9000, \quad \delta = 4.9, \text{ from } T = 3.0 \text{ to } 3.5, \\ \gamma = 18000, \quad \delta = 4.9, \text{ from } T = 3.5 \text{ to } 4.0. \end{cases}$$

The trajectory for upper ending point is

$$\begin{cases} \left[x_{upper}(T), y_{upper}(T) \right] = \left[T, 1 \right], \text{ from } T = 0 \text{ to } 1.0, \\ \left[x_{upper}(T), y_{upper}(T) \right] = \left[T, -1.6T^3 + 7.2T^2 - 9.6T + 5 \right], \text{ from } T = 1.0 \text{ to } 2.0, \\ \left[x_{upper}(T), y_{upper}(T) \right] = \left[T, 44.4444T^3 - 286.6667T^2 + 613.3333T - 433.7556 \right], \text{ from } T = 2.0 \text{ to } 2.3, \\ \left[x_{upper}(T), y_{upper}(T) \right] = \left[T, -0.1628T^3 + 1.5388T^2 - 4.4942T + 5.3777 \right], \text{ from } T = 2.3 \text{ to } 4.0. \end{cases}$$

The trajectory for lower ending point is

$$\begin{cases} \left[x_{lower}(T), y_{lower}(T) \right] = \left[T, 0 \right], \text{ from } T = 0 \text{ to } 3.0, \\ \left[x_{lower}(T), y_{lower}(T) \right] = \left[T, -0.1T^3 + 1.3T^2 - 5.1T + 6.3 \right], \text{ from } T = 3.0 \text{ to } 4.0. \end{cases}$$

The simulation result shown for *Panderichthys* in Fig. 7 is inverted vertically relative to the above.

Tiktaalik

The final time is T=5.50

$$\begin{cases} x'(T) = \sigma_x (x - vT) + v, \\ y'(T) = \left[y(0) - y_{lower}(0) \right] \dfrac{y'_{upper}(T) - y'_{lower}(T)}{y_{upper}(0) - y_{lower}(0)} + y'_{lower}(T), \\ \sigma_x = -0.2027, \quad v = 1.0. \end{cases}$$

$$\begin{cases} \gamma = 1500, \quad \delta = 4.7, \text{ from } T = 0 \text{ to } 0.5, \\ \gamma = 1000, \quad \delta = 4.7, \text{ from } T = 0.5 \text{ to } 2.0, \\ \gamma = 1500, \quad \delta = 4.9, \text{ from } T = 2.0 \text{ to } 3.5, \\ \gamma = 2700, \quad \delta = 4.9, \text{ from } T = 3.5 \text{ to } 4.5, \\ \gamma = 10000, \quad \delta = 4.9, \text{ from } T = 4.5 \text{ to } 5.0, \\ \gamma = 3000, \quad \delta = 4.9, \text{ from } T = 5.0 \text{ to } 5.5. \end{cases}$$

The trajectory for upper ending point is

$$\begin{cases} \left[x_{upper}(T), y_{upper}(T) \right] = \left[T, 1 \right], \text{ from } T = 0 \text{ to } 1.5, \\ \left[x_{upper}(T), y_{upper}(T) \right] = \left[T, -T^3 + 6T^2 - 11.25T + 7.75 \right], \text{ from } T = 1.5 \text{ to } 2.5, \\ \left[x_{upper}(T), y_{upper}(T) \right] = \left[T, 1.5 \right], \text{ from } T = 2.5 \text{ to } 4.5, \\ \left[x_{upper}(T), y_{upper}(T) \right] = \left[T, -0.5T^2 + 4.5T - 8.625 \right], \text{ from } T = 4.5 \text{ to } 5.5. \end{cases}$$

The trajectory for lower ending point is

$$\begin{cases} \left[x_{lower}(T), y_{lower}(T) \right] = \left[T, 0 \right], \text{ from } T = 0 \text{ to } 0.5, \\ \left[x_{lower}(T), y_{lower}(T) \right] = \left[T, 0.0741T^3 - 0.4444T^2 + 0.3889T - 0.0926 \right], \text{ from } T = 0.5 \text{ to } 3.5, \\ \left[x_{lower}(T), y_{lower}(T) \right] = \left[T, 0.25T^2 - 1.75T + 2.0625 \right], \text{ from } T = 3.5 \text{ to } 5.5. \end{cases}$$

The simulation result shown for *Tiktaalik* in Fig. 7 is inverted vertically relative to the above.
